# Severe delayed postpolypectomy bleeding in elder patient with post-polycythemia myelofibrosis

**DOI:** 10.1186/1471-2482-13-S2-S18

**Published:** 2013-10-08

**Authors:** Fabrizio Cardin, Maria Luigia Randi, Marco Mosele, Claudio Terranova, Carmelo Militello

**Affiliations:** 1 Geriatric Surgery Unit, Geriatrics Department, University Hospital of Padova, via Giustiniani n.2 Padova, Italy; 2 Department of Medical and Surgical Sciences, University Hospital of Padova, via Giustiniani n.2 Padova, Italy; 3 Section of Legal Medicine, Department of Molecular medicine, University Hospital of Padova, via Falloppio n.50, 35121 Padova, Italy

## Abstract

**Background:**

The interest of the case lies in an unexpected delayed bleeding following an endoscopic procedure in a patient with post-polycythemia myelofibrosis. The case gives the opportunity to discuss the medical management and monitoring of patients with myeloproliferative disorders undergoing minimally invasive surgery interventions.

**Case presentation:**

A 75 years old woman affected by post-polycythemia myelofibrosis underwent endoscopy polypectomy followed by a delayed major local bleeding. At the time of the endoscopy followed by bleeding, the platelet count was 837 × 109/L, haemoglobin 113 g/L, PCV 35,2% and WBC 20.22 × 106/L. No antithrombotic prophylaxis with low molecular weight heparin was used. Antiplatelet drug was withdraw seven days before endoscopy and restarted one week after the procedure. Polyp size was 11x19 mm and it was located on right side of the colon.

Fourteen days after procedure the patient developed a severe lower intestinal bleeding, which required RBC transfusion; the bleeding was in the site of polypectomy as demonstrated by arteriography; selective embolization of the three branches of the ileo-colic artery resolve the haemorrhage.

**Conclusion:**

There are some patients in whom current guidelines do not apply and our case stress the importance of myeloproliferative neoplasms as a risk factor for complications of endoscopic polypectomy. The delayed haemorrhage we observed suggest to strictly control the patient for a period longer than only one week also in case of antithrombotic treatment with antiplatelet drugs.

## Introduction

Endoscopic procedures are known to be a possible cause of significant or uncontrolled bleeding. High-risk procedures are considered those which have a risk of major bleeding higher than 1% [1].

The National Cancer Institute Common Terminology Criteria for Averse Events [2] defined as major bleeding those requesting hospitalization, transfusion support or a new surgical intervention. Polypectomy is generally considered a high-risk procedure: the overall incidence of clinically significant post-polypectomy bleeding may range from 0.2 to 1.6/1000 in large scale reports [3].

Haemorrhage may occur during (immediate bleeding) or some after (delayed bleeding) the procedure. Usually, the delayed bleeding which complicates 0.3-0.6% of polypectomies [4] occurs 3-7 days after the procedure, even if a haemorrhage occurred 29 days after polypectomy as been described [5].

The use of antithrombotic drugs complicates the management of gastrointestinal bleeding: in particular, post-endoscopic haemorrhages have been reported to occur in 6.1/1000 patients treated with aspirin [3]. However, recent guidelines [1] state that no adjustment in anticoagulation or antiplatelet therapy need to be made for low-risk procedures, irrespective of the underlying condition. In particular cases, antiplatelet drug withdraw 7-10 days before and resumed next day the procedure should be indicated, but the evidence is low [1]. While resuming anticoagulant therapy is associated with increased risk of severe delayed post-polypectomy bleeding, this seems not be the case with aspirin [6]. The larger polyp and those on the right site of the colon are those at higher risk of bleeding [7]. Other factors may also influence the risk of bleeding after polypectomy. Older age has been shown to be associated with increased risk [8] requesting increased risk of transfusion. Moreover, a special attention has to be given in some particular diseases, i.e. hemostatic myeloproliferative neoplasms, which have "per se" an increased risk of mucosal bruising. However, there are limited data assessing the bleeding risk of specific procedures in these settings and specifically no data have been found in patients with chronic myeloproliferative neoplasms (MPN). These diseases mainly affect people in middle-advanced age and many cases are diagnosed after 70 years.

The interest of the case lies in an unexpected delayed bleeding following an endoscopic procedure in a patient with post-polycythemia myelofibrosis. The case gives the opportunity to discuss the medical management and monitoring of patients with myeloproliferative neoplasms undergoing minimally invasive surgery interventions.

## Case presentation

A 75 years old woman underwent endoscopy polypectomy followed by a delayed major local bleeding. The history of the patients was characterized by polycythemia vera diagnosed in 1998. The patient was regularly followed in a haematological committed center for myeloproliferative neoplasms and treated with phlebotomies, low-dose aspirin and hydroyurea. In 2006 the presence of JAK2V617F mutation was demonstrated (allele burden 97%). The year later, increased WBC, presence of some immature circulating myeloid cells and a slight decrease in haemoglobin were observed together with an increased spleen volume suggesting a transformation into myelofibrosis. The clinical hypothesis was confirmed [9] with the observation of leukoerithroblastic peripheral blood picture and fibrosis grade 3 (on scale 0-3) in the bone marrow biopsy specimen,. Moreover, the CD34 cells in blood were 35 × 106/L and the cytogenetic study, which was not obtained at the time of diagnosis, showed a complex caryotype with del 5 and many break points in 5q3 and 5q33.

The endoscopy followed by bleeding was performed in 2008 when the platelet count was 837 × 109/L, haemoglobin 113 g/L, PCV 35,2% and WBC 20.22 × 106/L. No antithrombotic prophylaxis with LMWH was used. Antiplatelet drug was withdraw seven days before endoscopy and restarted one week after the procedure. Polyp size was 11x19 mm and it was located on right side of the colon.

Fourteen days after procedure the patient developed a severe lower intestinal bleeding, which required RBC transfusion; the bleeding was in the site of polypectomy as demonstrated by arteriography; selective embolization of the three branches of the ileo-colic artery resolve the haemorrhage. It is noteworthy that in 2010 the patient performed an aortic valve substitution complicated by a local hemorrhagic complication which requested again erythrocytes transfusion.

## Discussion

Myeloproliferative neoplasms (MPN) are clonal haematological diseases, interesting mainly median-advanced age, characterized by low rate of transformation into myelofibrosis and acute leukaemia but high risk of venous and arterial thrombosis and of haemorrhages [10]. There is a common belief that surgery could present an important circumstantial risk factor for thrombosis and bleeding [10], however, there are few available data on the incidence of these complications after surgical intervention in patient with MPN. On our best knowledge, no information is available regarding the risk of bleedings after endoscopic surgical procedures. The retrospective study from the GIMEMA group [11] on 311 surgical procedures in patients with polycythemia vera (PV) and essential thrombocythemia (ET) shows that the overall incidence of bleeding after surgery is of about 10 %, with a clear trend in subjects receiving antithrombotic prophylaxis and the hemorrhagic risk strongly related to the first 2 weeks of post-surgical period. Most of the patients experienced major haemorrhages that required transfusion treatment and prolonged hospital admission.

The use of aspirin to prevent thrombotic complications, which are the main causes of morbidity and mortality in MPN, is largely shared [12] but it is a common belief that aspirin has to be withheld one week before elective surgical procedures. The patients must be strictly followed for unpredictable development of thrombotic or hemorrhagic derangement. In our patient, despite the antiplatelet drug has been resumed 8 days after the procedure, a major bleeding occurred. We want to draw attention on the "window" period that follows endoscopic polypectomy. The delayed haemorrhage we observed suggest to strictly control the patient for a longer period than only one week. There are some patients in whom current guidelines do not apply and our case stress the importance of MPN as a risk factor for complications of endoscopic polypectomy. These evaluation should be considered also in medico-legal context where claims of malpractice could arise in case of thrombotic or haemorrhagic complications [13].

## Conclusion

In conclusion, in patients using antiplatelet drugs, surgeries involving mucous tissues, i.e. endoscopy polypectomy, has to be taken in due account because they may represent a relevant cause of delayed bleeding as the present case report shows.

## Consent

Written informed consent was obtained from the patient for publication of this case report. A copy of the written informed consent is available for review by the Editor-in-Chief of this journal.

## Competing interests

The authors declare that they have no competing interests.

## Authors' contributions

FC gave his contribution to the treatment, follow up and monitoring of the patient, to the literature review, to the drafting and reviewing of the paper, MLR analyzed and interpreted the patient data regarding the myeloproliferative disorder, MM assisted the patient, performed the review of the literature and gave his important contribution to writing the case report, CT contributed to analysis and interpretation of the data and to the writing of the paper, CM gave his contribution to the treatment and follow up of the patient. All the authors read and approved the final manuscript.

## Declarations

Publication of this article was funded by University Hospital of Padova, Italy.

This article has been published as part of *BMC Surgery *Volume 13 Supplement 2, 2013: Proceedings from the 26th National Congress of the Italian Society of Geriatric Surgery. The full contents of the supplement are available online at http://www.biomedcentral.com/bmcsurg/supplements/13/S2
